# Clinical presentation and radiologic imaging findings of phyllodes tumors: benign and borderline/malignant phyllodes tumors

**DOI:** 10.12688/f1000research.145872.2

**Published:** 2024-05-28

**Authors:** Wanrudee Lohitvisate, Kanitta Rodjanakonkiat, Amolchaya Kwankua

**Affiliations:** 1 Radiology, Thammasat University, Bangkok, Bangkok, 12120, Thailand

**Keywords:** Phyllodes, Breast tumors, Imaging features

## Abstract

**Background:**

Phyllodes tumor is a rare fibroepithelial neoplasm of the breast, which is classified histologically as benign, borderline, or malignant. Accurate preoperative diagnosis allows the correct surgical planning and reoperation avoidance.

**Objective:**

To describe the clinical presentation and radiologic features of phyllodes tumors and differentiate between benign and non-benign (borderline and malignant) groups.

**Methods:**

A retrospective study of 57 patients with a diagnosis of phyllodes tumor who had preoperative imaging (mammography, ultrasound, or CT chest) and histological confirmation. The data was collected from 1 June 2011 to 30 September 2021. The imaging features of the phyllodes tumors were described according to the 5th edition of the ACR BI-RADS lexicon. For comparing between two groups, the student t-test, Wilcoxon rank sum test, Chi-square test, and Fisher’s exact test were used for statistical analyses. The logistic regression analysis was calculated for non-benign phyllodes tumor prediction.

**Results:**

From 57 patients, the pathologic results were benign for 43 cases and non-benign phyllodes tumors for 14 cases. There was no differentiation of mammographic and CT features between benign and non-benign groups. Non-benign phyllodes tumors had the statistical significance of menopausal status, entire breast involvement, tumor size larger than 10 cm, and heterogeneous echo on univariable analysis. After multivariable analysis, menopausal status (odd ratios=13.79, p=0.04) and presence of vessels in the rim (odd ratios=16.51, p=0.019) or absent vascularity (odd ratios=8.45, p=0.047) on doppler ultrasound were significantly increased possibility of non-benign phyllodes tumor.

**Conclusions:**

Menopausal status and presence of vessels in the rim or absent vascularity on Doppler ultrasound were important predictors for the diagnosis of non-benign phyllodes tumor.

## Introduction

Phyllodes tumor is a rare fibroepithelial neoplasm of the breast. Most of phyllodes tumor are found in women between the age of 35 and 55 years. However, there are some reported cases of phyllodes tumor in young adult and elderly women. ^1^

In 2019, World Health Organization (WHO) classified phyllodes tumor into benign, borderline, and malignant based on several histologic features, including stromal cellularity, nuclear atypia, mitotic activity, stromal overgrowth, and tumor margin. ^2^^,^^3^ The majority of phyllodes tumors have been classified as benign (35% to 64%), and the remainder were the borderline and malignant subtypes. ^4^

The most common clinical presentation is a palpable breast mass that may be rapidly growing. ^1^ While an incidentally found mass on routine screening mammography are rare. ^5^ On physical examination, most patients have a smooth, round, well-defined, firm, painless and movable mass. The large masses may be associated with dilated veins visible over the skin, which may be stretched and attenuated. Nipple retraction, skin ulceration, invasion of the chest wall, and bloody nipple discharge have been reported but rare. Palpable enlarged axillary lymph nodes can be found up to 20% of patients but nodal metastasis of axillary lymph nodes is rare. ^6^

The mammographic features of phyllodes tumor are (i) circumscribed or oval mass (ii) a radiolucent halo may be seen around the lesion due to compression of the surroundings, and (iii) coarse calcifications may be present. ^4^^,^^7^^–^^9^ Ultrasonographic features show oval, circumscribed, echogenic rim, and hypoechoic mass. Fluid-filled clefts in a predominantly solid mass are highly suggestive of phyllodes tumor with good thorough transmission and lack of microcalcification. ^4^^,^^7^^,^^10^

Surgery has been the mainstay of treatment for all subtypes of phyllodes tumor. ^3^ Treatment of phyllodes tumor requires complete removal of the tumor with wide margins if the tumor is small and the simple mastectomy may require in the large tumor. ^11^ Furthermore, excision with negative margins should be taken for recurrent and malignant phyllodes tumors. ^12^

Because of accurate preoperative diagnosis allows the correct surgical planning and avoidance of reoperation, the purposes of this study are to describe clinical presentation and radiologic features of phyllodes tumors and differentiate between benign and non-benign (borderline and malignant) groups.

## Methods

### Study population

The data of female patients with histopathologically confirmed phyllodes tumor who had undergone preoperative mammography or ultrasonography or post contrast CT chest at Thammasat University Hospital from 1 June 2011 to 30 Sep 2021 was collected and retrospectively reviewed. The CT chest was performed in some patients due to the huge breast mass that cannot perform mammography and ultrasound.

The inclusion criteria were as follows; (a) patient who underwent breast surgery and received a histologic diagnosis of phyllodes tumor (b) the pre-operative radiological images (digital mammography or ultrasonography or post contrast CT chest) were available on Pictures Archiving and Communications System (PACS) of the Thammasat University Hospital, and (c) the medical records and pathological results were accessible on the information system of the Thammasat University Hospital (EPHIS). The exclusion criteria were as follows; (a) incomplete medical record and (b) inconclusive pathological results such as fibroepithelial tumor.

Of the initial 70 suspicious phyllodes tumors, 10 cases were excluded because of incomplete medical record and unavailable pre-operative images, and 3 cases had inconclusive pathological result as fibroepithelial tumors from core needle biopsy and no other procedure to receive more tissue. Eventually, a total of 57 phyllodes tumors were eligible for our study. The final pathologic results of phyllodes tumors were benign for 43 cases and non-benign (borderline and malignant) for 14 cases.

### Imaging technique and processing

The patients were received at least one of the imaging studies as follows;

(a) Mammographic imaging was performed by using digital technique of Lorad Selenia (Hologic) or 3-Dimensions (Hologic) mammography on mediolateral oblique (MLO) and craniocaudal (CC) views.
(b) Ultrasonography (US) was performed with B-mode grayscale and doppler mode on supine position using a Samsung RS80A equipped with an L3-12A linear transducer (3–12 MHz, 5.0 cm), or a Philips IU22 equipped with an L12-5 linear transducer (5–12 MHz, 5.0 cm).
(c) Pre and post contrast enhanced computed tomography (CT) of the chest using Siemens or Phillips – 128/256 slices model.

### Clinicopathological evaluation

The patient’s clinical information was collected from electronic medical records consist of age at diagnosis, clinical presentation (palpable mass, breast pain, skin ulcer, or screening), duration of symptom, menstrual status, side of tumor (right or left), location of the tumor, treatment (wide excision or mastectomy), and metastasis (none, lymph node, or distant).

The histopathological data were retrieved from the final histopathological reports which were separated into two groups: benign and non-benign (borderline and malignant) phyllodes tumor. The phyllodes tumor was categorized based on histopathological features, using World Health Organization (WHO) criteria which divided histologic features of phyllodes tumor based on stromal atypia, mitosis, stromal overgrowth, and margin. ^2^

### Imaging interpretation

The images (mammography, ultrasonography, or contrast enhanced CT chest) were retrospectively reviewed by two radiologists with 13 years and 12 years of experience in breast imaging. For discrepant results, a final consensus was reached after discussion. The images were randomly selected and blinded to histopathological results.

The images were retrieved retrospectively from PACS. The latest images before undergoing treatment were selected and reviewed. The details of each imaging modality were described as follows;

(a) Mammography: The data included side (right or left), size (in largest dimension) and morphology of the phyllodes tumor by using the fifth edition ACR BI-RADS lexicon. The mammographic features were identified as presence of calcification (macro-, micro-, or none), shape (irregular, oval, round), margin (circumscribed, obscured, microlobulated, indistinct, spiculated), density (high, equal, low, fat-containing), location (upper outer, upper mid, upper inner, mid outer, mid inner, lower outer, lower mid, lower inner), and involvement (<1quadrant, 1-2 quadrant, >2quadrants).
(b) Ultrasonography: The data included side (right or left), size (in largest dimension), morphology and vascularity of the phyllodes tumor by using the fifth edition ACR BI-RADS lexicon. The ultrasonographic features were identified as shape (irregular, oval, round), margin (circumscribed, indistinct, angular, microlobulated, spiculated), echogenicity (hypo-, hyper-, iso-, heterogeneous), posterior features (no posterior features, enhancement, shadowing, combined pattern), cystic area (present, absent), and vascularity (absent, internal vascularity, vessels in rim)
(c) Contrast enhanced CT chest: Using the same data in the mammographic features and consider the enhancement of the phyllodes tumor in Hounsfield Unit (HU) as follows ^1^: No enhancement; the difference of the phyllodes tumor’s attenuation between pre- and post-contrast administration was less than 10 HU, ^2^ Enhancement: the difference of the phyllodes tumor’s attenuation between pre- and post-contrast administration was equal or greater than 10 HU, and degree of enhancement was classified as mild enhancement (the enhancement was less than 25%), moderate enhancement (the enhancement was 25-50%), and marked enhancement (the enhancement was greater than 50%).

### Data analysis and statistics

The demographic data of the patients and histopathological results of all phyllodes tumors were reported by using the number (percentage) for categorical variables and mean ± SD or median (IQR) and range for continuous variables.

To compare the clinicopathological and imaging characteristics between benign and non-benign phyllodes tumor, we initially used Shapiro-wilk normality test before using Student’s t-test for normally distributed continuous variables and Wilcoxon rank sum test for non-normally distributed continuous variables. The Chi-square test or Fisher’s exact test was used for the categorical variables. All statistical analyses were performed with R program (version 4.1.1, R foundation for Statistical Computing, Vienna, Austria) and statistical significance will be considered as p-value less than 0.05.

With regard to the data analyses, the demographic data and imaging features were discovered to be statistically significant; logistic regression analysis was conducted to calculate the odds ratios (OR) with 95% confidence intervals (95% CI) for predicting non-benign phyllodes tumor probability as compared with benign phyllodes tumor. Odds ratios were contemplated to indicate statistical difference if the 95% CI excluded 1.0. A univariate analysis was first performed to identify any potential predictor variables. The demographic data and imaging features with a p-value <0.05 according to a univariate analysis were included in the multivariate analysis to determine any independent predictors of the non-benign phyllodes tumor. A stepwise selection procedure was used to identify variables with a p-value <0.05.

## Results

### Patient characteristics and histopathological results

A total of 57 female patients with a mean age of 38.8 years (range, 15-66 years) were included in the study. Forty-three patients were benign, 7 patients were borderline, and 7 patients were malignant subtype. The demographic data, clinical characteristics, and histopathological results of patients are shown in Table 1. ^22^

**Table 1.** Demographic and clinical characteristics of patients.

	Benign (n=43)	Non-benign (n=14)	Total (n=57)	P-value
Age				0.078
Mean (SD)	37.1 (12.0)	43.9 (13.6)	38.8 (12.7)	
Range	15-66	24-62	15-66	
Clinical presentation, n (%) ( #)				
Palpable mass	40 (93)	12 (85.7)	52 (91.2)	0.587
Breast pain	12 (27.9)	1 (7.1)	13 (22.8)	0.152
Skin ulcer	0	2 (14.3)	2 (3.5)	0.057
Screening	2 (4.7)	2 (14.3)	4 (7)	0.25
Menstrual status, n (%)				0.009 *
Pre-menopause	34 (79)	6 (42.9)	40 (70.2)	
Menopause	6 (14)	7 (50.0)	13 (22.8)	
Unknown	3 (7)	1 (7.1)	4 (7.0)	
Duration (months) ( ##)				0.991
Median (25-75% IQR)	4 (2-12)	5 (1.9-9)	4 (2-12)	
Range	0.03-24	0.25-60	0.03-60	
Side, n (%)				0.701
Right	23 (53.5)	6 (42.9)	29 (50.9)	
Left	20 (46.5)	8 (57.1)	28 (49.1)	
Location, n (%)				
Upper				
Outer	9 (20.9)	2 (14.3)	11 (19.3)	0.714
Mid	5 (11.6)	1 (7.1)	6 (10.5)	1
Inner	8 (18.6)	1 (7.1)	9 (15.8)	0.427
Mid				
Outer	1 (2.3)	2 (14.3)	3 (5.3)	0.146
Inner	2 (4.7)	0 (0)	2 (3.5)	1
Subareolar	4 (9.3)	0 (0)	4 (7)	0.563
Lower				
Outer	5 (11.6)	0 (0)	5 (8.8)	0.319
Mid	1 (2.3)	0 (0)	1 (1.8)	1
Inner	1 (2.3)	1 (7.1)	2 (3.5)	0.434
Entire breast	7 (16.3)	7 (50)	14 (24.6)	0.027 *
Treatment, n (%)				0.271
Wide excision	35 (81.4)	9 (64.3)	44 (77.2)	
Mastectomy	8 (18.6)	5 (35.7)	13 (22.8)	
Metastasis, n (%)				0.057
None	43 (100)	12 (85.7)	55 (96.5)	
Lymph node	0 (0)	0 (0)	0 (0)	
Distant	0 (0)	2 (14.3)	2 (3.5)	

^#^ Some patients had multiple symptoms at the time of presentation.

^##^ Duration of the patients who presented with mass on screening images was excluded for statistical analysis.

* Statistically significant at p value < 0.05 determined by Fisher’s exact test.

There was no statistically significant difference in patient’s age, clinical presentation, duration, side, treatment or metastasis between benign and non-benign groups. The mean ages of patients with benign and non-benign phyllodes tumors were 37.1 years and 43.9 years, respectively. Palpable breast mass was the most common (91.2%) presenting symptom in both groups. Noted that in some patients can had more than one symptom at the time of presentation. The median duration of symptoms prior to diagnosis was 4 months, which ranged from 0.03 month (1 day) to 60 months (5 years). For the patients whose phyllodes tumor incidentally found on screening were not included for statistical analysis. Both sides of breast were equally affected from both benign and non-benign phyllodes tumors.

Most patients (77.2%) from both groups underwent wide excision of the tumor as a treatment. The duration of the first imaging procedure to surgery were ranged from 1 day to 610 days and the median duration was about 110 days. Time interval between imaging procedure and surgery of malignant Phyllodes tumors were 1 day to 31 days. Some patients who were benign or borderline Phylloes tumor, had time interval more than 6 months because of significantly increased size of tumor during imaging follow up. There were 2 distant metastatic patients (14.3%) from the non-benign group which both of them were lung metastasis. None of the non-benign phyllodes tumor patients had lymph node metastasis.

There were statistically significant differences in the menstrual status (p=0.009) and location of the tumor (p=0.027) between benign and non-benign phyllodes tumors. The majority of patients in benign group were pre-menopausal status (79%), while 50% of the patients in non-benign group were menopausal status (p=0.009). Tumor involving entire breast was more presented in patients with non-benign phyllodes tumor (p=0.027).

### Mammographic features

Thirty-one patients underwent mammography. The patients who did not undergo mammography due to 1) the tumor size was too large to be performed mammography, and 2) patients were younger than 30 years old, so they were undergoing ultrasonography for the evaluation instead of mammography. However, our study did not show any statistically significant differences in all mammographic features between the two groups as demonstrated in Table 2. Noted that of all 31 patients, 7 patients were excluded from size evaluation because the masses were obscured by surrounding dense breast parenchyma.

**Table 2.** Comparison of mammographic features.

	Benign (n=26)	Non-benign (n=5)	Total (n=31)	P-value
Side, n (%)				0.344
Right	13 (50)	1 (20)	14 (45.2)	
Left	13 (50)	4 (80)	17 (54.8)	
Size, n (%)( #)				1
<5 cm	12 (60)	3 (75)	15 (62.5)	
5-10 cm	5 (25)	1 (25)	6 (25)	
>10 cm	3 (15)	0 (0)	3 (12.5)	
Calcification, n (%)				NA
Macrocalcification	0 (0)	0 (0)	0 (0)	
Microcalcification	0 (0)	0 (0)	0 (0)	
None	26 (100)	5 (100)	31 (100)	
Shape				1
Irregular	7 (36.8)	2 (50)	9 (39.1)	
Oval	8 (42.1)	1 (25)	9 (39.1)	
Round	4 (21.1)	1 (25)	5 (21.7)	
Border, n (%)				0.251
Circumscribed	16 (80)	2 (50)	18 (75)	
Obscured	3 (15)	1 (25)	4 (16.7)	
Microlobulated	0 (0)	0 (0)	0 (0)	
Indistinct	1 (5)	1 (25)	2 (8.3)	
Spiculated	0 (0)	0 (0)	0 (0)	
Density, n (%)				1
High density	12 (60)	2 (50)	14 (58.3)	
Equal density	8 (40)	2 (50)	10 (41.7)	
Low density	0 (0)	0 (0)	0 (0)	
Fat density	0 (0)	0 (0)	0 (0)	
Location, n (%)				
Upper				
Outer	4 (20)	2 (50)	6 (25)	0.251
Mid	1 (5)	0 (0)	1 (4.2)	1
Inner	2 (10)	0 (0)	2 (8.3)	1
Mid				
Outer	0 (0)	0 (0)	0 (0)	-
Inner	1 (5)	0 (0)	1 (4.2)	1
Subareolar	3 (15)	0 (0)	3 (12.5)	1
Lower				
Outer	1 (5)	0 (0)	1 (4.2)	1
Mid	1 (5)	0 (0)	1 (4.2)	1
Inner	2 (10)	1 (25)	3 (12.5)	0.437
Entire breast	5 (25)	1 (25)	6 (25)	1
Involvement, n (%)				1
<1 quadrant	8 (40)	2 (50)	10 (41.7)	
1-2 quadrant	7 (35)	1 (25)	8 (33.3)	
>2 quadrant	5 (25)	1 (25)	6 (25)	

^#^ Seven patients were excluded because of dense breast.

### Ultrasonographic features

All 57 patients with phyllodes tumor underwent ultrasonography. There was no statistically significant difference in the tumor’s side, shape, border, posterior acoustic features, and cystic area between benign and non-benign groups as demonstrated in Table 3.

**Table 3.** Comparison of ultrasonographic features.

	Benign (n=43)	Non-benign (n=14)	Total (n=57)	P-value
Side, n (%)				0.701
Right	23 (53.5)	6 (42.9)	29 (50.9)	
Left	20 (46.5)	8 (57.1)	28 (49.1)	
Size, n (%) (#)				0.038 *
<5 cm	30 (70)	6 (43)	36 (63)	
5-10 cm	9 (21)	1 (7)	10 (18)	
>10 cm	4 (9)	7 (50)	11 (19)	
Shape				0.28
Irregular	25 (58.1)	11 (78.6)	36 (63.2)	
Oval	15 (34.9)	2 (14.3)	17 (29.8)	
Round	3 (7)	1 (7.1)	4 (7)	
Border, n (%)				0.19
Circumscribed	32 (74.4)	7 (50)	39 (68.4)	
Not circumscribed				
Indistinct	3 (7)	3 (21.4)	6 (10.5)	
Angular	1 (2.3)	0 (0)	1 (1.8)	
Microlobulated	7 (16.3)	4 (28.6)	11 (19.3)	
Spiculated	0 (0)	0 (0)	0 (0)	
Echogenicity, n (%)				0.019 *
Hypoechogenicity	34 (79.1)	6 (42.9)	40 (70.2)	
Hyperechogenicity	0 (0)	0 (0)	0 (0)	
Isoechogenicity	1 (2.3)	0 (0)	1 (1.8)	
Heterogeneous echo	8 (18.6)	8 (57.1)	16 (28.1)	
Posterior acoustic features, n (%)				0.524
Shadowing	1 (2.3)	0 (0)	1 (1.8)	
Enhancement	14 (32.6)	8 (57.1)	22 (38.6)	
Mixed feature	2 (4.7)	2 (14.3)	4 (7.0)	
None	19 (44.2)	4 (28.6)	23 (40.4)	
Cystic area, n (%)				1
Present	29 (67.4)	9 (64.3)	38 (66.7)	
Absent	14 (32.6)	5 (35.7)	19 (33.3)	
Vascularity, n (%)				0.04 *
Absent	4 (9.3)	3 (21.4)	7 (12.3)	
Internal vascularity	37 (86)	8 (57.1)	45 (78.9)	
Vessels in rim	2 (4.7)	3 (21.4)	5 (8.8)	

* Statistically significant at p value < 0.05 determined by Fisher’s exact test.

**Figure 1.**
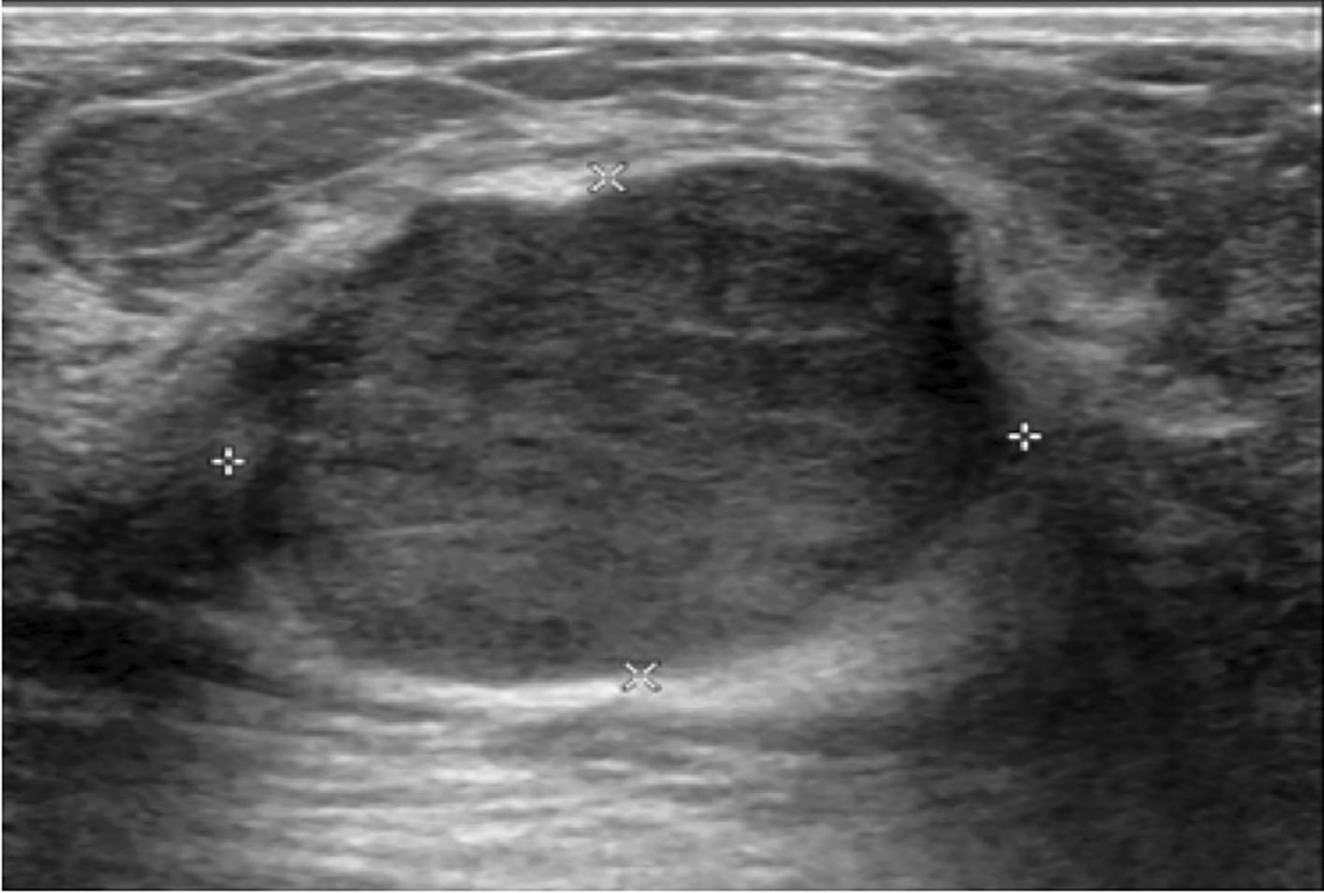
A 15-year-old girl with a palpable left breast mass for six months. The ultrasound showed a 3.0x2.3x3.3-cm well-defined lobulated hypoechoic mass with posterior acoustic enhancement at the left 12 o’clock. Wide excision of the mass was done, and the histopathologic result was a benign phyllodes tumor.

**Figure 2.**
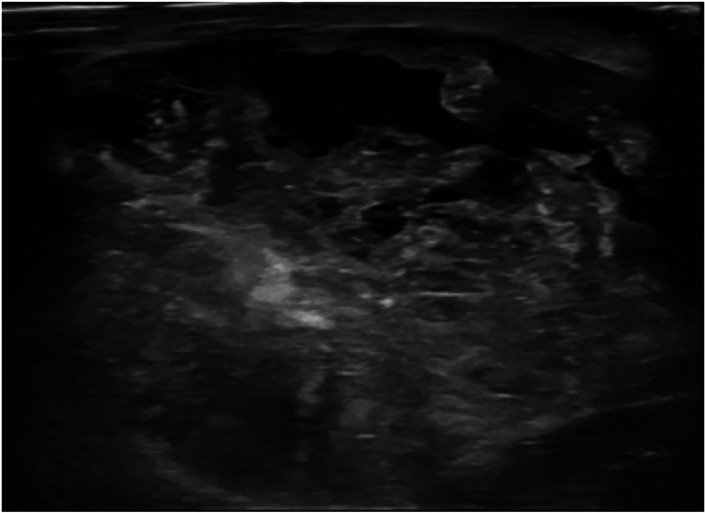
A 42-year-old woman with malignant phyllodes tumor. The ultrasound showed a huge circumscribed heterogeneous echoic mass with internal cystic spaces involving the entire right breast.

**Figure 3.**
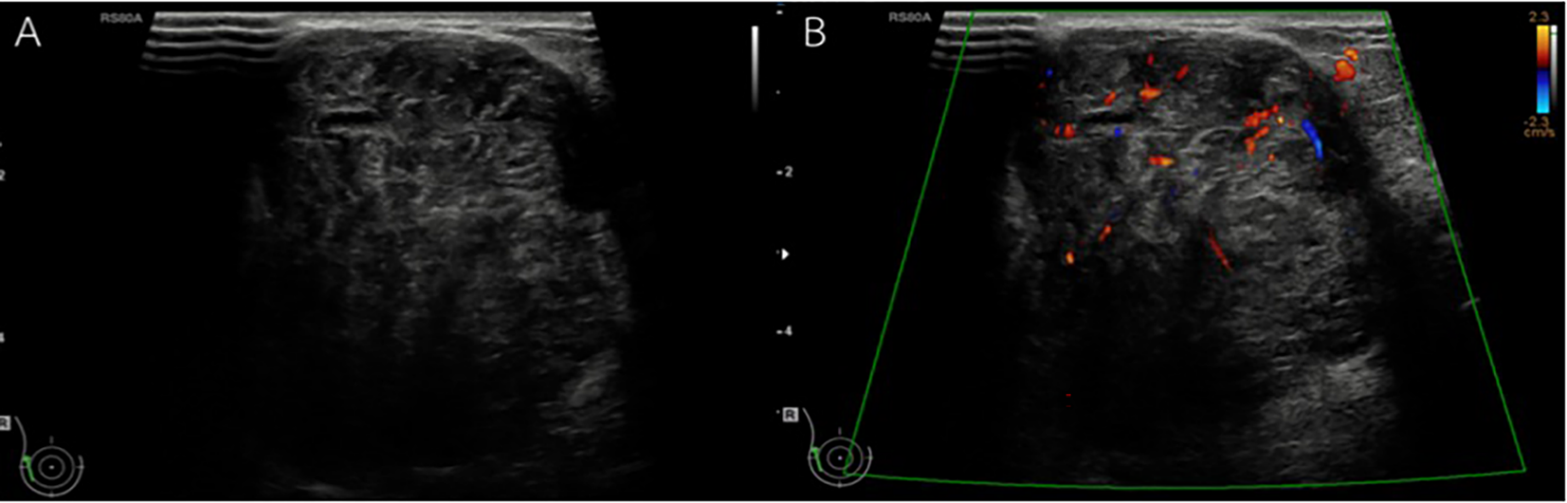
A 62-year-old woman with palpable right breast mass for one year. A. The gray-scale ultrasound showed a large, partially well-defined, heterogeneous hypoechoic mass with small internal cystic spaces involving the outer region of the right breast and B. The color doppler ultrasound showed internal vascularity in the mass. The histopathologic result was a benign phyllodes tumor.

There were statistically significant differences in the size (p= 0.038), echogenicity (p= 0.019) and vascularity (p= 0.04) of phyllodes tumors between benign and non-benign groups. Size of the tumors was commonly less than 5 cm in both benign and non-benign groups (30 of 43 [70%] vs 6 of 14 [43%], respectively). Whereas the tumor size more than 10 cm was more common in non-benign than those of benign group (7 of 14 [50%] vs 4 of 43 [9%], respectively). The most common echogenicity of benign phyllodes tumors was hypoechogenicity ( Figure 1). Whereas the heterogeneous echogenicity ( Figure 2) was higher found in non-benign phyllodes tumor compared to benign phyllodes tumor (8 of 14 [57.1%] vs 8 of 43 [18.6%], respectively). None of the phyllodes tumors had hyperechogenicity on ultrasonographic images. The most common vascularity characteristics in both benign and non-benign phyllodes tumors was presence of internal vascularity (37 of 43 [86%] vs 8 of 14 [57.1%], respectively) ( Figure 3).

Univariable analysis ( Table 4), using the logistic regression to analyze the correlation between demographic data and ultrasonographic features to predict non-benign phyllodes tumor, the results showed that patients with menopausal status (p= 0.008), tumor located in entire breast (p = 0.015), tumor size more than 10 cm (p= 0.029) and heterogeneous echo on ultrasound images (p= 0.009) were significantly related with non-benign phyllodes tumor.

**Table 4.** Logistic regression analysis for predicting non-benign phyllodes tumor.

Variable	Univariate		Multivariate	
	OR (95% CI)	P value	OR (95% CI)	P value
Menstrual status				
Pre-menopause (R)	1		1	
Menopause	6.61 (1.64,26.64)	0.008 *	13.79 2.31,82.24)	0.004 *
Location				
Other location (R)	1		-	
Entire breast	5.14 (1.37,19.33)	0.015 *		
Ultrasonographic features				
Size		0.037 *		
< 5 cm (R)	1		-	
5-10 cm	0.56 (0.06,5.24)	0.608		
>10 cm	15 (1.32,169.87)	0.029 *		
Echogenicity				
Hypoechogenicity (R)	1		-	
Heterogeneous echo	5.67 (1.53,20.98)	0.009 *		
Vascularity		0.077		0.016 *
Internal vascularity (R)	1		1	
Absent	1.47(0.65,18.63)	0.147	8.45 (1.03,69.15)	0.047 *
Vessels in rim	6.94 (0.99,48.55)	0.051	16.51(1.59,171.96)	0.019 *

* Statistically significant at p value 0.05.

(R) = Reference.

After multivariable analysis ( Table 4) with multicollinearity avoidance with stepwise selection procedure, menopausal status (odd ratios, 13.79 [95% CI: 2.31,82.24], p value of 0.04), and presence of vessels in rim (odd ratios, 16.51 [95% CI: 1.59,171.96], p value of 0.019) or absent vascularity on doppler ultrasound (odd ratios, 8.45 [95% CI: 1.03,69.15], p value of 0.047) were significantly increased possibility of non-benign phyllodes tumor. But the location, tumor size and echogenicity showed no statistically significant difference for predicting non-benign phyllodes tumor.

### CT features

There were only 5 (8.8%) from 57 phyllodes tumor patients who underwent contrast enhanced CT chest. One (20%) patient was benign and 4 (80%) patients were non-benign phyllodes tumors. Because of the small number of subjects, we would only describe CT findings without statistical analysis as demonstrated in Table 5.

**Table 5.** Comparison of CT features.

	Benign (n=1)	Non-benign (n=4)	Total (n=5)
Side, n (%)			
Right	1 (100)	2 (50)	3 (60)
Left	0 (0)	2 (50)	2 (40)
Size, n (%)			
<5 cm	0 (0)	0 (0)	0 (0)
5-10 cm	0 (0)	1 (25)	1 (20)
>10 cm	1 (100)	3 (75)	4 (80)
Calcification, n (%)			
Macrocalcification	0 (0)	0 (0)	0 (0)
Microcalcification	0 (0)	1 (25)	1 (20)
None	1 (100)	3 (75)	4 (80)
Shape			
Irregular	1 (100)	4 (100)	5 (100)
Oval	0 (0)	0 (0)	0 (0)
Round	0 (0)	0 (0)	0 (0)
Border, n (%)			
Circumscribed	0 (0)	3 (75)	3 (60)
Not circumscribed			
Irregular	1 (100)	1 (25)	2 (40)
Spiculated	0 (0)	0 (0)	0 (0)
Density, n (%)			
Homogeneous	0 (0)	0 (0)	0 (0)
Heterogeneous	1 (100)	4 (100)	5 (100)
Enhancement, n (%)			
No enhancement	0 (0)	2 (50)	2 (40)
Enhancement			
Mild (<25%)	0 (0)	0 (0)	0 (0)
Moderate (25-50%)	1 (100)	0 (0)	1 (20)
Marked (>50%)	0 (0)	2 (50)	2 (40)
Location, n (%)			
Entire breast	1 (100)	4 (100)	5 (100)
Involvement, n (%)			
<1 quadrant	0 (0)	0 (0)	0 (0)
1-2 quadrant	0 (0)	0 (0)	0 (0)
>2 quadrant	1 (100)	4 (100)	5 (100)

**Figure 4.**
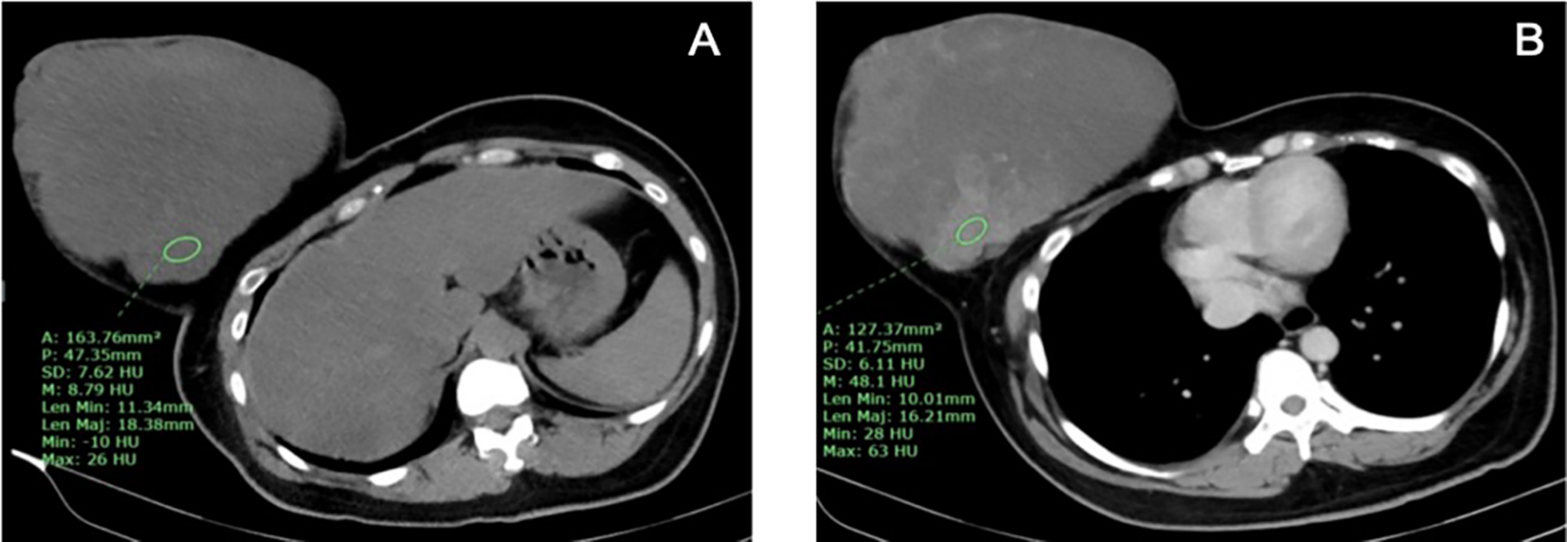
A 37-year-old woman with borderline phyllodes tumor. A. The pre-contrast CT chest showed an irregularly shaped, heterogeneous hypodense mass involving the entire right breast with skin invasion and B. After contrast administration, the tumor showed marked heterogeneous enhancement.

The one benign phyllodes tumor was found in the right breast, while the non-benign phyllodes tumors were found in each side of the breasts equally. The size of phyllodes tumor ranged from 8.9 to 24.5 cm in the longest dimension, which both of the smallest and the largest tumors were found in non-benign group. Only 1 non-benign phyllodes tumor showed microcalcification, while the rest of phyllodes tumors did not present calcification. All of phyllodes tumors were irregular shape, heterogeneous density, involving more than 2 quadrants and located in entire breast. The border of phyllodes tumor was circumscribed in 3 (75%) patients from non-benign group whereas irregular border was found in 1 (100%) patient from benign group and 1 (25%) patient from non-benign group. There were 2 (50%) non-benign phyllodes tumors that showed no enhancement, while the other 2 (50%) non-benign phyllodes tumor had marked enhancement ( Figure 4), and 1 (100%) benign phyllodes tumor showed moderate enhancement.

## Discussion

Phyllodes tumor is a rare fibroepithelial neoplasm of the breast that accounts for 0.3% to 0.5% of breast tumors in female which is classified histologically as benign, borderline, or malignant. Accurate preoperative diagnosis allows correct surgical planning and avoidance of reoperation.

According to the prior studies, most of phyllodes tumors were benign subtype. ^3^^,^^13^^,^^14^ Similarly, the majority of phyllodes tumors (75.4%) in our study were benign.

There was no statistically significant difference of the patient’s age between the two groups but the mean age was higher in non-benign phyllodes tumor group (43.9 years). Our study also found patients younger than 30 years old in both groups. Palpable mass was the most common clinical finding in both benign and non-benign groups. These data could be found in patients with fibroadenoma but usually occur younger than phyllodes tumor. To differentiate fibroadenoma from benign phyllodes tumor, we suggest combining the age of patient and the rate of tumor growth. Fibroadenomas are found in younger age group and slower rate of tumor growth than phyllodes tumor. However, it might be difficult to diagnose the juvenile fibroadenoma which occurs in adolescent patients and have rapid growth of tumor. In this case, tissue biopsy for histopathologic results is helpful.

There was statistically significant difference of tumor size in ultrasound images which was higher in non-benign phyllodes tumor. Most of tumor size in benign group (70%) and almost half of non-benign group (43%) was less than 5 cm. The duration of symptom was no statistically significant difference. Therefore, the rate of tumor growth especially in small tumor cannot help to differentiate malignant from benign phyllodes tumor in our study. Nevertheless, some studies revealed rapid growth may be detected in malignant tumors. ^15^^,^^16^

Kılıç MÖ et al. found that the most cases of phyllodes tumor were diagnosed in premenopausal women. ^15^ Our study showed similar results of the benign group and all of phyllodes tumor. But menopausal status was found statistically significance (p= 0.009) in the non-benign group. Furthermore, after multivariate analysis, we found that menopausal status was the important predictor for non-benign phyllodes tumor (p=0.004).

Few prior studies showed that the upper outer quadrant of the breast was the most frequent location of phyllodes tumor, and both sides were equally affected. ^17^^,^^18^ In our study, there was no predilection side of breast to be affected by phyllodes tumor in both benign and non-benign groups. The location of phyllodes tumor was slightly higher in the upper outer quadrant for both groups, whereas tumor involving the entire breast was more frequently found in non-benign group (p= 0.027). Kılıç MÖ et al found a small number of patients that had both multifocality and bilaterality of phyllodes tumors. ^15^ However, all of the patients in our study had only a single tumor. A case that had more than one phyllodes tumor was excluded from the study due to incomplete medical record.

The mammographic findings in our study were parallel to the other literature that there was no specific radiologic feature to differentiate the histologic subtypes of phyllodes tumor. ^15^

Kalambo M et al. showed that irregular lesion and the longest dimension of the tumor greater than 7 cm on ultrasound were the significant univariate predictors of increased risk of borderline or malignant phyllodes tumors. ^19^ Some prior studies showed no significant difference of vascularity between the different subtypes of phyllodes tumors. ^13^^,^^20^^,^^21^ Even though, in our study most of morphologic features on ultrasound were not statistically significant difference between groups. Non-benign phyllodes tumor had irregular shape, indistinct and microlobulated borders more often than benign phyllodes tumor. The univariable analysis of our study revealed statistical significance of menopausal status, entire breast involvement, tumor size larger than 10 cm and heterogeneous echo of tumor in non-benign phyllodes tumor. Furthermore, after multivariate analysis, we found that menopausal status and vessels in rim/absent vascularity on doppler ultrasound were significantly increased possibility of non-benign phyllodes tumor (p=0.004 and 0.047/0.019, respectively). The stromal overgrowth with hypercellularity and internal areas of necrosis or hemorrhage might be causes of absent vascularity or only present of peripheral vascularity on doppler ultrasound in the malignant phyllodes tumor and some large tumors.

Five patients with large and huge tumor (size ranged from 8.9 to 24.5 cm in longest dimension) which involved the entire breast underwent post contrast enhanced CT chest and did not undergo mammography. The pathologic results were benign for 1 case and borderline/malignant for 4 cases. All of them had irregular shape and heterogeneous density. Non-benign phyllodes tumor had marked contrast enhancement for 50% and had internal microcalcifications for 25%. Due to the very small number of subjects, we did not demonstrate statistical analysis for characteristic features of the phyllodes tumors between these groups. However, the huge mass with irregular shape and marked contrast enhancement might be prefer non-benign phyllodes tumor. Some invasive ductal carcinomas (IDC) may present with large or huge breast mass with abnormal skin changes that cannot be differentiated from malignant phyllodes tumor clinically. Our study found that there was no nodal metastasis or associated calcifications from mammographic images in all borderline/malignant phyllodes tumors. Therefore, we suggest that the huge, non-calcified breast mass without axillary lymphadenopathy would prefer malignant phyllodes tumor to IDC.

The patients with non-benign phyllodes tumor were treated by mastectomy for 36% due to very large tumor size and had distant metastasis for 14%, therefore, prediction of borderline/malignant phyllodes tumor has affected for treatment option of each patient.

## Conclusion

Most of patients with phyllodes tumor were presented with palpable breast mass. Menopausal status and presence of vessels in rim or absent vascularity on doppler ultrasound were important predictors for borderline/malignant phyllodes tumor. There was no mammographic or CT features to differentiate between benign and non-benign phyllodes tumors.

### Limitations

There were some limitations in this study. First, the study was a retrospective design study that may have had a selection bias. Second, this was single-centered study that may cause a relatively small sample size and an unequal number of patients between benign and non-benign phyllodes tumor. Multicentered study with a larger population and data sets may be needed to validate our findings.

## Ethics and consent

The Human Research Ethics Committee of Thammasat University (Medicine) approved conducting this research with the certificate project number MTU-EC-RA-0-285/64, and approved on December 30, 2021. The inform-consent was waived requirement due to the retrospective nature of the study.

## Data Availability

Zenodo: Clinical Presentation and Radiologic Imaging Findings of Phyllodes Tumors: Benign and Borderline/Malignant Phyllodes Tumors. https://zenodo.org/doi/10.5281/zenodo.10538896. ^22^ This project contains the following underlying data:
- Demographic phyllodes study.xlsx (demographic data of this study) - MMG-CT-US findings phyllodes project.xlsx (imaging findings of this study) Data are available under the terms of the Creative Commons Attribution 4.0 International license (CC-BY 4.0).

## References

[1] Reinfuss M Mituś J Duda K : The treatment and prognosis of patients with phyllodes tumor of the breast: an analysis of 170 cases. *Cancer.* 1996;77(5):910–916. 10.1002/(SICI)1097-0142(19960301)77:5<910::AID-CNCR16>3.0.CO;2-6 8608483

[2] The WHO Classification of Tumours Editorial Board: *WHO Classification of Tumours: Breast Tumours.* 5th ed. Lyon, France: International Agency for Research on Cancer (IARC);2019.

[3] Abdul Hamid S Rahmat K Ramli MT : Radiopathological characteristics and outcomes of phyllodes tumor of the breast in Malaysian women. *Medicine (Baltimore).* 2018;97(31):e11412. 10.1097/MD.0000000000011412 30075507 PMC6081195

[4] Mishra SP Tiwary SK Mishra M : Phyllodes tumor of breast: a review article. *ISRN Surg.* 2013;2013:1–10. 10.1155/2013/361469 PMC3615633 23577269

[5] Mangi AA Smith BL Gadd MA : Surgical management of phyllodes tumors. *Arch. Surg.* 1999;134:487–493. discussion. 10.1001/archsurg.134.5.487 10323420

[6] Telli ML Horst KC Guardino AE : Phyllodes tumors of the breast: natural history, diagnosis, and treatment. *J. Natl. Compr. Cancer Netw.* 2007;5(3):324–430. 10.6004/jnccn.2007.0027 17439760

[7] Feder JM Paredes ES de Hogge JP : Unusual breast lesions: radiologic-pathologic correlation. *Radiographics.* 1999;19:S11–S26. 10.1148/radiographics.19.suppl_1.g99oc07s11 10517440

[8] Jorge Blanco A Vargas Serrano B Rodriguez Romero R : Phyllodes tumors of the breast. *Eur. Radiol.* 1999;9:356–360. 10.1007/s003300050680 10101663

[9] Cosmacini P Veronesi P Zurrida S : Mammography in the diagnosis of phyllodes tumors of the breast. Analysis of 99 cases. *Radiol. Med.* 1991;82(1-2):52–55. 1654580

[10] Cole Beuglet C Soriano R Kurtz AB : Ultrasound, X-ray mammography, and histopathology of cystosarcoma phylloides. *Radiology.* 1983;146(2):481–486. 10.1148/radiology.146.2.6294737 6294737

[11] Muttarak M Lerttumnongtum P Somwangjaroen A : Phyllodes tumour of the breast. *Biomed. Imaging Interv. J.* 2006;2(2):e33. 10.2349/biij.2.2.e33 21614232 PMC3097610

[12] Tan BY Acs G Apple SK : Phyllodes tumours of the breast: a consensus review. *Histopathology.* 2016;68(1):5–21. 10.1111/his.12876 26768026 PMC5027876

[13] Chao T-C Lo Y-F Chen S-C : Phyllodes tumors of the breast. *Eur. Radiol.* 2003;13(1):88–93. 10.1007/s00330-002-1370-x 12541114

[14] Liberman L Bonaccio E Hamele-Bena D : Benign and malignant phyllodes tumors: mammographic and sonographic findings. *Radiology.* 1996;198(1):121–124. 10.1148/radiology.198.1.8539362 8539362

[15] Kılıç MÖ Terzioğlu SG Bozkurt B : Phyllodes tumor of the breast: Analysis of 48 patients. *J. Breast Health.* 2016;12(4):158–164. 10.5152/tjbh.2016.3100 28331755 PMC5351441

[16] Atalay C Kınaş V Çelebioğlu S : Analysis of patients with phyllodes tumor of the breast. *Ulus Cerrahi Derg.* 2014;30:129–132. 10.5152/UCD.2014.2719 25931913 PMC4379857

[17] Confavreux C Lurkina A Mitton N : Sarcomas and malignant phyllodes tumours of the breast – A retrospective study. *Eur. J. Cancer.* 2006;42:2715–2721. 10.1016/j.ejca.2006.05.040 17023158

[18] Barrio AV Clark BD Goldberg JI : Clinicopathologic features and long-term outcomes of 293 phyllodes tumors of the breast. *Ann. Surg. Oncol.* 2007;14:2961–2970. 10.1245/s10434-007-9439-z 17562113

[19] Kalambo M Adrada BE Adeyefa MM : Phyllodes tumor of the breast: ultrasound-pathology correlation. *AJR.* 2018 Apr;210:W173–W179. 10.2214/AJR.17.18554 29412020

[20] Spitaleri G Toesca A Botteri E : Breast phyllodes tumor: a review of literature and a single center retrospective series analysis. *Crit. Rev. Oncol. Hematol.* 2013;88:427–436. 10.1016/j.critrevonc.2013.06.005 23871531

[21] Umpleby H Moore I Royle G : An evaluation of the preoperative diagnosis and management of cystosarcoma phyllodes. *Ann. R. Coll. Surg. Engl.* 1989;71:285–288. 2552895 PMC2499001

[22] Lohitvisate W Rodjanakonkiat K Kwankua A : Clinical Presentation and Radiologic Imaging Findings of Phyllodes Tumors: Benign and Borderline/Malignant Phyllodes Tumors (1/2023).[Dataset]. *Zenodo.* 2024. 10.5281/zenodo.10538897 PMC11153989 38845824

