# Supplementary material for: Clinical presentation and radiologic imaging findings of phyllodes tumors: benign and borderline/malignant phyllodes tumors
Source: F1000Res. 2024 May 28;13:210. Originally published 2024 Mar 21. [Version 2] doi: 10.12688/f1000research.145872.2 (PMC11153989; doi:10.12688/f1000research.145872.2)
Supplement: Peer review history

# Reviewer report: Reviewer response for version 2

**DOI:** 10.5256/f1000research.166872.r283575

**Published:** 2024-06-05

**Authors:** Jatuporn Chayakulkheeree

**Affiliations:** 1 Chulalongkorn University, Bangkok, Thailand

This revised article is sufficient quality to be considered for indexing now

Is the work clearly and accurately presented and does it cite the current literature?

Yes

If applicable, is the statistical analysis and its interpretation appropriate?

I cannot comment. A qualified statistician is required.

Are all the source data underlying the results available to ensure full reproducibility?

Yes

Is the study design appropriate and is the work technically sound?

Yes

Are the conclusions drawn adequately supported by the results?

Yes

Are sufficient details of methods and analysis provided to allow replication by others?

Yes

Reviewer Expertise:

Breast imaging, Diagmostic Radiology

I confirm that I have read this submission and believe that I have an appropriate level of expertise to confirm that it is of an acceptable scientific standard.

# Reviewer report: Reviewer response for version 1

**DOI:** 10.5256/f1000research.159888.r260117

**Published:** 2024-05-21

**Authors:** Jatuporn Chayakulkheeree

**Affiliations:** 1 Chulalongkorn University, Bangkok, Thailand

Overall, the research is well-conducted and provides valuable insights into the clinical and radiologic characteristics of phyllodes tumors. Here are some key points for the author team to consider.

 1. please provide information about the time interval between imaging and surgery. This is important for understanding the relevance of the imaging findings to the surgical intervention and final pathology.

 2. please condense the discussion part.

 Hopefully, with some minor revisions to enhance clarity and provide additional detail, the manuscript could make a valuable contribution.

Is the work clearly and accurately presented and does it cite the current literature?

Yes

If applicable, is the statistical analysis and its interpretation appropriate?

I cannot comment. A qualified statistician is required.

Are all the source data underlying the results available to ensure full reproducibility?

Yes

Is the study design appropriate and is the work technically sound?

Yes

Are the conclusions drawn adequately supported by the results?

Yes

Are sufficient details of methods and analysis provided to allow replication by others?

Yes

Reviewer Expertise:

Breast imaging, Diagmostic Radiology

I confirm that I have read this submission and believe that I have an appropriate level of expertise to confirm that it is of an acceptable scientific standard, however I have significant reservations, as outlined above.

# Reviewer report: Reviewer response for version 1

**DOI:** 10.5256/f1000research.159888.r260123

**Published:** 2024-04-18

**Authors:** Kobkun Muangsomboon

**Affiliations:** 1 Siriraj hospital, Mahidol University, Siriraj, Thailand

The paper is good and advantage for health science. It was conducted with the proper methodology, statistics and ethical issue. Please let me note some opinions to authors team for consideration. That might be beneficial for clinical application. 

 - The title of the paper says about the imaging findings, it is better if the authors can provide Mammographic images in the cases that underwent both mammogram and ultrasound. It will be complete information. And also that can help learning for the people who are interested in this article. (although, the paper found that mammographic features are no difference between benign and non-benign group) If the number of images is not over the journal limitation.

 - The time between the imaging and surgery in the paper should be stated.(may be range or less than 1 month, or etc.)

 - The information of the size of the tumour incidentally found should be added in the result.(range or detail...)

 - In table 2, the marker should be placed in the row of shape, border , density, location (same as size)

 - In the discussion part, the last paragraph shows the percent of the reason of mastectomy. It is the data of this paper or from the reference, please cite. If the data is the result of the paper (please give the short information of metastasis in the text)

 -I just have a question that in a group of only biopsy (not excision), the pathology report form biopsy may not represent the character of the whole breast lesion. (Based on the available demographic data, 8 are biopsies only and the pathology showed 7 benign, 1 borderline). In my practice, if the biopsy showed benign result, the follow up image (ultrasound) should be performed to confirm the benignity (stable in size and internal characteristic). So I asked for a post-biopsy follow up period for the benign group. If the lesions were stable (in a period of time that authors can provide) , the clinical and images findings were analyzed in the correct group. If the lesion changed in size (or rapid growth) during the follow up period, the re-biopsy or excision should be performed (may be not true benign?) If the authors can state that all benign in only biopsy cases were stable for a period of time. It will be better and no question for readers.

Is the work clearly and accurately presented and does it cite the current literature?

Yes

If applicable, is the statistical analysis and its interpretation appropriate?

Yes

Are all the source data underlying the results available to ensure full reproducibility?

Yes

Is the study design appropriate and is the work technically sound?

Yes

Are the conclusions drawn adequately supported by the results?

Yes

Are sufficient details of methods and analysis provided to allow replication by others?

Yes

Reviewer Expertise:

-

I confirm that I have read this submission and believe that I have an appropriate level of expertise to confirm that it is of an acceptable scientific standard.
